# A cross-sectional study of testosterone deficiency and inflammatory markers in older men

**DOI:** 10.3389/fendo.2025.1606949

**Published:** 2025-07-31

**Authors:** Iwona Rotter, Żaneta Ciosek, Anna Syroka, Aleksandra Ryl

**Affiliations:** Department of Medical Rehabilitation and Clinical Physiotherapy, Pomeranian Medical University, Szczecin, Poland

**Keywords:** testosterone, aging men, Hs-CRP, hormonal, deficiency

## Abstract

**Background:**

This cross-sectional study aimed to examine the relationship between total testosterone (TT) levels, the diagnosis of testosterone deficiency syndrome (TDS), and high-sensitivity C-reactive protein (hsCRP) concentrations in aging men. The analysis also included selected hormonal and anthropometric parameters.

**Methods:**

Serum hsCRP levels were measured. Additionally, serum levels of TT, estradiol (E2), dehydroepiandrosterone sulfate (DHEA-S), insulin (I), and sex hormone-binding protein (SHBG) were assessed using ELISA. Patients were divided based on the presence or absence of a TDS diagnosis.

**Results:**

In patients without TDS, no significant correlation was observed between hsCRP levels and other measured variables. However, higher hsCRP levels were associated with an increased BMI, larger waist and hip circumferences, and elevated triglyceride (TAG) levels compared to patients with lower hsCRP concentrations.

**Conclusions:**

The co-occurrence of testosterone deficiency and elevated inflammatory markers such as hsCRP was associated with less favorable metabolic and anthropometric profiles. While causality cannot be inferred from this observational study, the findings suggest a possible link between systemic inflammation and testosterone deficiency in aging men. These associations merit further investigation in longitudinal and mechanistic studies to clarify directionality and underlying biological pathways.

## 1 Introduction

Testosterone is the primary male sex hormone and androgen in males. Testosterone is synthesized in 95% in the testes and to a much lesser extent in the adrenal cortex. Besides its action on the male reproductive system, testosterone nonspecifically intensifies overall metabolism and affects muscle mass and strength, fat distribution, bone mass, and erythropoiesis (1). However, there is no doubt that age-related changes in the function of internal secretion organs play a significant role in the aging process, especially in men, where age-related reduction in gonadal androgen production leads to testosterone deficiency and the so-called testosterone deficiency syndrome (TDS). It can significantly deteriorate the quality of life and negatively affect the function of many organs and systems (1).

C-reactive protein (CRP) levels, a marker of inflammation, may be inversely correlated with testosterone levels in men (2). This relationship could have significant clinical implications, as both low testosterone levels and chronic inflammation are associated with numerous health issues in men, including an increased risk of cardiovascular disease, erectile dysfunction, decreased libido, osteoporosis, and depression. Gaining insight into the pathways connecting CRP and testosterone could support the creation of new treatments for individuals with testosterone deficiency and chronic inflammatory conditions. The complex interplay between testosterone, inflammation, and disease is strongly modulated by adipose tissue, which enhances aromatase activity – an enzyme responsible for the conversion of testosterone into estradiol. This enzymatic transformation inhibits the hypothalamic–pituitary axis, thereby diminishing testosterone synthesis (3). Visceral adipose tissue acts as a metabolically active organ that secretes inflammatory mediators such as cytokines, adipokines, and various pro-inflammatory agents, including IL-6, IL-1β, TNF-α, and plasminogen activator inhibitor-1, all of which contribute to both systemic and localized vascular inflammation and impairment. Moreover, adipose tissue releases leptin, which suppresses the hypothalamic–pituitary–gonadal axis by interfering with gonadotropin signaling in Leydig cells, resulting in reduced androgen production (4). While these mechanistic pathways are supported by the secretion profile of visceral adipose tissue, it should be noted that the present analysis primarily relied on hsCRP as the inflammatory marker. Although hsCRP is a widely used surrogate of systemic inflammation, it may not fully capture the complexity of the inflammatory milieu associated with obesity-related hypogonadism. In particular, cytokines such as IL-6, TNF-α, and adipokines like leptin could provide additional mechanistic insights and better reflect tissue-specific inflammatory processes. Therefore, future studies should incorporate a broader panel of inflammatory markers to more accurately characterize the immunometabolic alterations underlying the pathophysiology of obesity-induced testosterone deficiency.

Testosterone plays a sig ificant ole in inhibiting adipose tissue formation and the expression of various adipocytokines, such as leptin, TNF-α, IL-6, and IL-1, and it is positively associated with adiponectin levels (5). A decline in testosterone secretion significantly contributes to changes in the body composition of aging males (6), marked by a decrease in fat-free mass and an increase in fat mass. Moreover, adipose tissue begins to release proinflammatory cytokines, leading to elevated serum leptin and CRP levels, coupled with decreased adiponectin levels (7, 8).

Low levels of testosterone, as well as high levels of C-reactive protein (CRP), have been linked to increased all-cause mortality risk in men (6, 9). Testosterone regulates cytokine expression through androgen receptors, modulating the inflammatory response (10). Studies suggest a bidirectional relationship between obesity-stimulated cytokine levels and TT (11). Obesity, comorbidities, and aging may play a key role in this relationship, contributing to androgen deficiency through the secretion of adipocytokines and CRP (10).

While the precise mechanism through which testosterone influences these responses remains uncertain, laboratory evidence suggests that testosterone has the ability to suppress proinflammatory cytokines while potentially up-regulating anti-inflammatory cytokines. Recognizing the association between androgens and inflammation is critical, given the pivotal role inflammation plays in the pathogenesis of numerous diseases. If a significant association is established, it could provide justification for considering androgen replacement therapy to enhance outcomes for patients with inflammatory disorders.

The literature on this topic exhibits a diversity of perspectives regarding the relationship between CRP and androgens in men and androgen levels. Numerous studies indicate the existence of a relationship between these two parameters; however, the results of these studies are often contradictory and dependent on various factors. Some studies suggest an inverse correlation between CRP and testosterone (4, 12). Conversely, other studies fail to corroborate such a distinct relationship (13, 14). The diversity of results from previous studies highlights the need for further research that considers both hormonal and anthropomorphic factors influencing this relationship. The aim of the study was to analyze the relationship between TT concentration and diagnosing TDS and hsCRP in aging men in relation to selected hormonal and anthropometric parameters.

## 2 Materials and methods

The study involved 313 male volunteers aged 50 to 75 years (mean age: 61.31 ± 6.33), recruited through primary care physicians in Szczecin, Poland, who provided comprehensive information about the study’s aims and procedures. Participation was contingent upon written, informed consent, which was obtained from all individuals prior to enrollment. Men were excluded if they had diabetes, were undergoing cancer treatment, used neuroleptics, antidepressants, or steroids, were receiving testosterone therapy, or had liver or thyroid disorders, ascites, or hernias located along the linea alba or in postoperative scars. The study protocol was approved by the Bioethics Committee of the Pomeranian Medical University in Szczecin (KB-0012/155/16) and followed the ethical principles of the Declaration of Helsinki for research involving human subjects.

Anthropometric measurements were conducted, including body mass, height, and abdominal circumference, with the calculation of body mass index (BMI) (15).

### 2.1 Collecting research material

Blood samples were drawn from the ulnar vein in fasted subjects between 7:30 am and 9:00 am. The blood was collected into tubes containing a clotting activator and gel separator, followed by centrifugation. Subsequently, the serum was stored at -70°C.

### 2.2 Determination of hormo al a d biochemical param ters

High sensitivity C-reactive protein levels in the serum were measured using a spectrophotometric method with ready-made reage t kits (Biolabo, Aqua-Med, Łódź, Poland). Additionally, serum concentrations of hormones such as total testostero e (TT), estradiol (E2), dehydroepiandrosterone sulfate (DHEA-S), insulin (I), and sex hormone binding protein (SHBG) were assessed via ELISA using commercially available reagent kits (DRG-MedTek, Warsaw, Poland). The tests were performed in a certified research laboratory that participates in the quality control program conducted by the Central Center for Quality Research in Laboratory Diagnostics (COJBwDL). The laboratory has a quality management system certificate IS0 9001:2008 and PN-EN 152224:2013.

### 2.3 Criteria for group division

**Figure 1:**
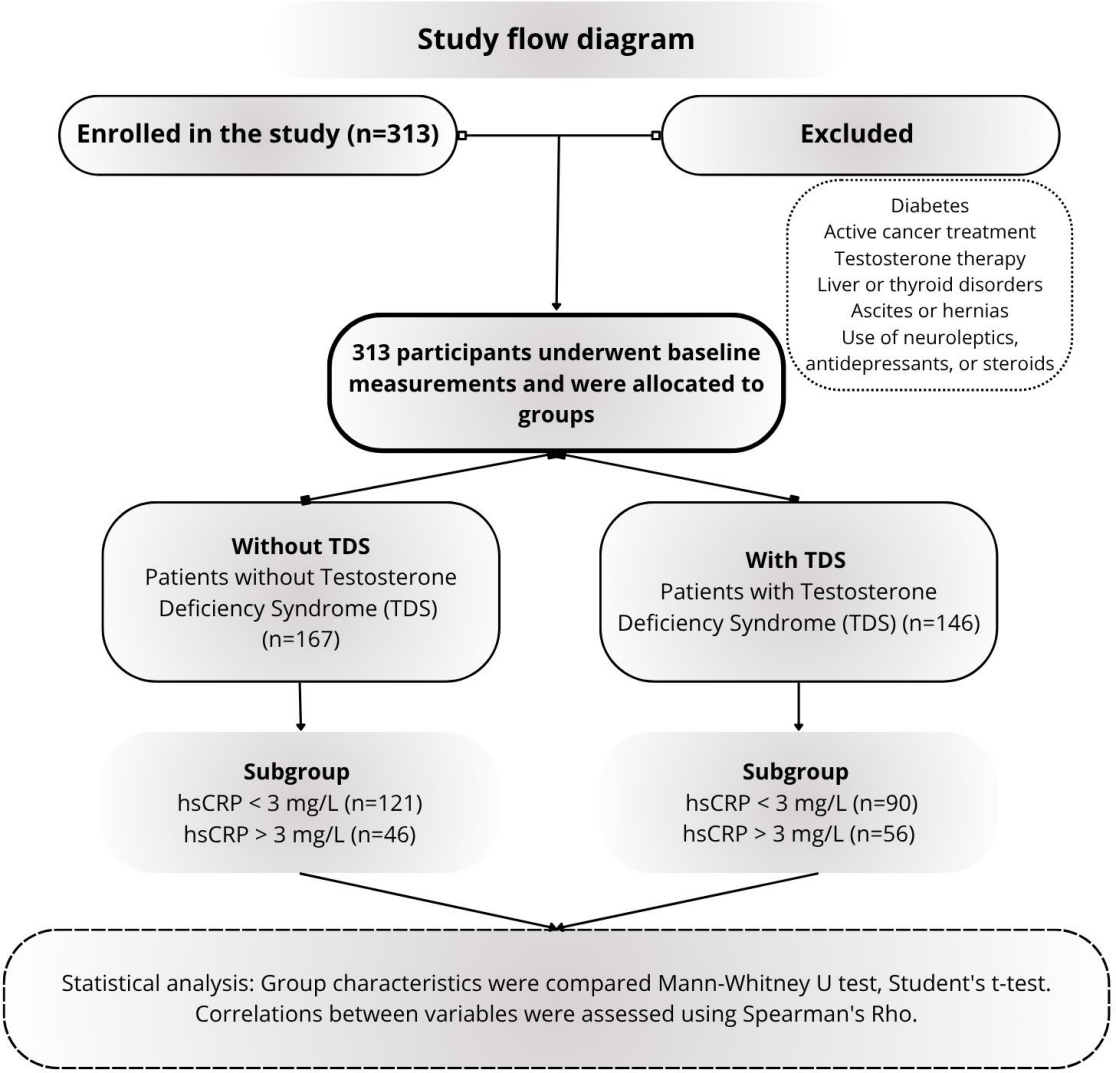
Design of the research conducted, taking into account the study groups; testosterone deficiency syndrome (TDS), TT, total testosterone; LH, luteinizing hormone; SHBG, sex hormone binding globulin; DHEA-S, dehydroepiandrosterone sulfate; E2, estradiol; I, Insulin; hsCRP, high sensitivity C-reactive protein.

The diagnosis of TDS syndrome adhered to the consensus recommendations established by the International Society of Andrology (ISA), International Society for the Study of the Aging Male (ISSAM), European Association of Urology (EAU), European Academy of Andrology (EAA), and American Society of Andrology (ASA) (16). Patients exhibiting total testosterone (TT) levels below 2.5 ng/ml or within the range of 2.5 ng/ml to 3.5 ng/ml, alongside clinical symptoms assessed using the Morley questionnaire (17), were categorized into the TT-deficient group. The study showed that 127 men with TDS showed concerns on the Morley adropause scale, whereas in the group of men without TDS, 3 men showed these symptoms. In the study, the cut -off value of hs CRP was considered to be 3 mg/l (18). The study design is presented in **Figure 1**.

### 2.4 Statistical analysis

The statistical analysis was car ied out utilizing the Statist ca 13.1 software. Within this analysis, the group’s characteristics were deli eated, showcasing m dian alues, means, and standard deviations, as well as minimum and maximum values. Normality assessm nts of distributions were conducted employing the Shapiro-Wilk test. Group compariso s were executed utilizing either the Mann-Whitney U test or the Student’s t-test. Additionally, Spearman’s Rho correlation analysis was conducted. Results with a p value less than or equal to 0.05 were considered statistically significant.

## 3 Results

The study investigated the correlation between anthropometric factors, hormone levels, and hsCRP concentrations in patients with and without testosterone deficiency (**Table 1**). The analysis highlighted statistically significant differences between the two groups concerning body mass, BMI, waist circumference, and hip circumference. Furthermore, variations were observed in testosterone (TT), sex hormone-binding globulin (SHBG), estradiol (E2), insulin (I), and hsCRP levels among these patients. In **Table 2**, the relationship between anthropometric factors, and hormone levels in the group of patients without testosterone deficiency was analyzed based on hsCRP concentration. The analysis did not show any correlation in the group of patients without TDS based on the level of hsCRP concentration. In **Table 3**, the relationship between anthropometric factors, and hormone levels in the group of patients with testosterone deficiency (TDS) was analyzed based on hsCRP concentration. It was indicated that patients with higher hsCRP levels exhibit a higher BMI, larger waist and hip circumferences, and higher triglyceride (TAG) levels compared to patients with lower hsCRP concentrations. The study also conducted an analysis examining the correlation between anthropometric and hormonal indicators and the hsCRP value (**Table 4**). Results revealed that among patients without TDS, a positive correlation exists between hsCRP value and waist circumference (R=0.155, p=0.045), alongside a negative correlation with total testosterone (R=-0.182, p=0.018). Conversely, within the TDS patient cohort, a positive association was observed between hsCRP value and body mass (R=0.213, p=0.010), BMI (R=0.300, p=0.001), hip circumference (R=0.275, p=0.001), and waist circumference (R=0.305, p=0.0001).

**Table 1:** Relationship between anthropometric factors, hormone levels, and hsCRP in patients with and without testosterone deficiency.

Variables	Patients without TDS, (n=167)					Patients with TDS, (n=146)					p
	X	Me	Min	Max	SD	X	Me	Min	Max	SD	
Age [years]	61.4	62.000	50.00	75.00	6.84	61.15	61.00	50.00	74.00	5.720.88	
Body weight [kg]	82.69	82.00	52.00	128.00	12.70	92.25	90.00	60.00	135.00	15.82	<0.001*
BMI [kg/m2]	26.95	26.79	19.49	42.28	3.63	29.82	29.06	20.02	46.71	4.63	<0.001*
Hip Circumference [cm]	101.26	101.00	82.00	129.00	7.08	105.61	105.00	87.00	127.00	7.75	<0.001*
Abdominal Circumference [cm]	98.51	98.00	75.00	133.00	10.43	106.20	104.00	79.00	146.00	12.48	<0.001*
TT [ng/ml]	4.91	4.53	2.50	9.83	1.51	2.66	2.70	0.55	3.49	0.60	<0.001*
LH [IU/ml]	8.32	7.36	1.08	40.44	4.56	7.60	6.83	1.57	33.09	4.15	0.097
SHGB [nmol/l]	53.51	49.89	11.65	192.00	26.34	35.85	35.59	3.94	99.05	17.57	<0.001*
DHEA-S [μg/ml]	1.34	1.23	0.01	4.24	0.80	1.42	1.27	0.05	4.61	0.88	0.502
E2 [pg/ml]	43.44	37.73	7.30	133.66	23.60	34.17	31.56	5.61	88.73	17.72	<0.001*
I [μmol/l]	13.49	12.27	1.22	66.42	7.56	16.58	16.21	2.33	35.61	7.46	<0.001*
hsCRP [mg/l]	4.24	1.70	0.03	43.77	6.94	6.04	2.79	0.10	90.41	10.51	0.004*

Testosterone deficie cy sy drome (TDS); BMI, body mass index; TT, total testosterone; LH, luteinizing hormone; SHBG, sex hormone binding globulin; DHEA-S, dehydroepiandrosterone sulfate; E2, estradiol; I, insulin; hsCRP, high se sitivity C, eactive protein, *Statistically significant parameter.

**Table 2:** Relationship between anthropometric factors, and hormone levels in the group of patients without testosterone deficiency according to hsCRP concentration.

Variables	Patients without TDS				Patients without TDS				p
	with hsCRP below 3 (n=121)				with hsCRP above 3 (n=46)				
	Me	SD	Min	Max	Me	SD	Min	Max	
BMI [kg/m2]	26.58	3.29	19.71	35.93	26.88	4.30	19.49	42.28	0.193
Hip circumference[cm]	101.00	6.69	82.00	121.00	103.00	7.93	88.00	129.00	0.105
LH [IU/ml]	7.33	3.66	1.08	20.53	7.44	6.42	2.77	40.44	0.685
SHGB [nmol/l]	49.86	22.35	13.84	128.80	54.05	34.15	11.65	192.00	0.882
DHEA-S [μg/ml]	1.20	0.75	0.09	4.17	1.24	1.01	0.03	4.24	0.799
E2 [pg/ml]	37.49	20.11	7.30	107.16	40.50	26.68	9.85	106.62	0.537
I [μmol/l]	11.96	6.23	1.22	31.93	12.04	10.87	3.78	66.42	0.257
TT [ng/ml]	4.70	1.50	2.53	9.83	4.22	1.61	2.51	8.69	0.182

Testosterone deficiency syndrome (TDS), BMI, body mass index; TT, total testosterone; LH, luteinizing hormone; SHBG, sex hormone binding globulin; DHEA-S, dehydroepiandrosterone sulfate; E2, estradiol; I, insulin; hsCRP, high sensitivity; C, reactive protein.

**Table 3:** The relationship between anthropometric factors, and hormone levels in the group of patients with testosterone deficiency syndrome (TDS) according to hsCRP concentration.

Variables	Patients with TDS				Patients with TDS				p
	with hsCRP below 3 (n=90)				with hsCRP above 3 (n=56)				
	Me	SD	Min	Max	Me	SD	Mini	Max	
BMI [kg/m2]	28.07	3.76	20.68	41.32	31.12	4.88	24.22	46.71	<0.001*
Abdominal circumference [cm]	101.00	10.61	79.00	137.00	110.00	12.24	92.00	146.00	<0.001*
Hip circumference[cm]	102.00	7.07	87.00	125.00	107.00	7.95	93.00	127.00	0.002*
LH [IU/ml]	6.98	4.40	1.57	33.09	7.20	3.62	2.34	22.70	0.797
SHGB [nmol/l]	37.60	18.38	3.94	84.31	34.04	16.87	5.17	99.05	0.197
DHEA-S [μg/ml]	1.30	0.74	0.18	3.47	1.32	0.91	0.05	3.51	0.690
E2 [pg/ml]	31.82	15.40	5.61	77.68	31.05	20.15	13.70	88.73	0.301
I [μmol/l]	18.47	6.64	2.33	33.58	13.54	8.02	6.07	35.61	0.426
TT [ng/ml]	2.77	0.55	1.07	3.49	2.65	0.55	1.12	3.49	0.524

Testosterone deficiency syndrome (TDS), BMI, body mass index; TT, total testosterone; LH, luteinizing hormone; SHBG, sex hormone binding globulin; DHEA-S, dehydroepiandrosterone sulfate; E2, estradiol; I, insulin; hsCRP, high sensitivity C-reactive protein, *Statistically significant parameter.

**Table 4:** Analysis of correlation between anthropometric and hormonal indicators and hsCRp value.

Correlations	Patients without TDS, n=167		Patients with TDS, n=146	
	R	p	R	p
Body mass	0.022	0.778	0.213	0.010*
BMI	0.121	0.121	0.300	0.001*
Hip circumference	0.054	0.488	0.275	0.001*
hsCRP				
Abdominal circumference	0.155	0.045*	0.305	0.001*
TT	-0.182	0.018*	-0.106	0.202
LH	0.017	0.826	-0.039	0.639
SHGB	-0.051	0.510	-0.124	0.136
DHEA-S	0.032	0.681	-0.043	0.603
E2	0.089	0.252	0.051	0.538

Testosterone deficiency syndrome (TDS), BMI, body mass index; TT, total testosterone; LH, luteinizing hormone; SHBG, sex hormone binding globulin; DHEA-S, dehydroepiandrosterone sulfate; E2, estradiol; I, Insulin; hsCRP, high sensitivity C, reactive protein, *Statistically significant parameter.

## 4 Discussion

In recent years, extensive research has delved into the connections between testosterone and the inflammatory process (19). However, the recognition of bidirectional mechanisms linking the immune and endocrine systems dates back at least two decades (20). Testosterone has been associated with the suppression of pro-inflammatory substances and promotion of anti-inflammatory cytokines, potentially contributing to reduced inflammation. Furthermore, assertions regarding the anti-inflammatory properties of testosterone stem from observations of heightened inflammatory cytokine levels in hypogonadal men and reductions in inflammatory markers observed in testosterone supplementation studies (20). Conversely, an inflammatory process, indicative of heightened oxidative stress, has been associated with negative influences on androgen levels (21). This relationship has been noted both through direct disruption of reproductive tissue and by impairing the regulatory mechanisms of the hypothalamic–pituitary–gonadal (HPG) axis.Testosterone deficiency syndrome is diagnosed when low serum testosterone levels coincide with clinical symptoms of hypogonadism. Research involving over 2,000 men in the United States indicated that nearly 39% of men aged over 45 exhibit biochemical indicators of testosterone deficiency (22). In contrast, a study involving 890 men showed that testosterone deficiency was present in 20% of individuals aged 60–89 years, 30% in those aged 70–79, and 50% in participants over 80 years old (23). These results underscore the widespread prevalence of testosterone deficiency in older men when considering biochemical markers.

In our research, higher hsCRP concentrations were associated with elevated BMI values, larger waist and hip circumferences, and higher TAG levels compared to patients with lower concentrations of this parameter. This relationship between hsCRP levels and anthropometric parameters such as body weight and abdominal circumference has been widely documented. For instance, Fernandes et al. (24) reported that individuals who maintained regular physical activity were less likely to exhibit elevated hsCRP levels. Specifically, high physical activity levels were associated with lower hsCRP in both men (OR=0.44 [0.30 to 0.65]) and women (OR=0.35 [0.16 to 0.76]). Among overweight or obese individuals and smokers, those who were constantly active also had a lower likelihood of elevated hsCRP levels compared to their physically inactive peers. Further evidence comes from studies involving individuals with schizophrenia, where BMI and hsCRP have been linked to abnormal lipid profiles (25). A direct association between BMI and hsCRP was observed across demographic variables such as age, sex, ethnicity, and education. Additionally, hsCRP showed an inverse relationship with HDL cholesterol in the overall sample and among those with overweight or obesity, but not in individuals of normal weight. These findings suggest that inflammation and dyslipidemia are more commonly associated with overweight and obesity in individuals with schizophrenia.

Similar conclusions were reached by Buljubasic et al. (26), who found that hsCRP levels may mediate the relationship between primary hypertension and overweight. However, it is important to recognize the limitations of BMI as a measure. While widely used, BMI does not distinguish between lean and fat mass, meaning that individuals with identical BMI values may have significantly different body compositions. For this reason, bioelectrical impedance analysis (BIA) is increasingly recommended as a more precise method for assessing body composition, including the proportions of fat and lean tissue. Moreover, our research findings suggest a positive correlation between hsCRP levels and waist circumference (R=0.155, p=0.045), and a negative correlation with TT (R=-0.182, p=0.018) in patients without TDS. In contrast, among patients with TDS, hsCRP levels were positively associated with body mass (R=0.213, p=0.010), BMI (R=0.300, p=0.001), hip circumference (R=0.275, p=0.001), and waist circumference (R=0.305, p=0.0001).

Tremellen et al. reported that reduced testosterone levels in men were significantly associated with elevated inflammatory markers, particularly in individuals with obesity (27). However, findings by Grandys et al. (2021) indicated that the relationship between testosterone and inflammation markers such as CRP and ferritin (FER) was influenced by body mass index and not independent of it (20). In a cross-sectional study by Maggio et al., involving 473 men over the age of 65 from the InCHIANTI cohort (a population-based study of older adults in the Chianti region of Tuscany, Italy), an inverse correlation was observed between testosterone levels and the concentration of soluble IL-6 receptor (sIL-6r) – a receptor fragment that may enhance IL-6 activity. No similar associations were found for other inflammatory markers. These findings suggest a close interplay between reduced testosterone levels and intensified pro-inflammatory activity in older males (28). Supporting this, Kaplan et al. also reported an inverse association between serum testosterone and high-sensitivity C-reactive protein (hsCRP) in elderly men (29). Other studies have similarly reported associations between low testosterone (hypogonadism) and elevated hsCRP and additional inflammatory markers in aging males (20, 30, 31).

A further prospective study identified a connection between elevated hsCRP levels and reduced concentrations of bioavailable testosterone in both cross-sectional and longitudinal analyses. Notably, this association remained significant after adjusting for potential confounders, and men with higher hsCRP levels were more likely to develop hypogonadism over a ten-year period. It is worth noting that some long-term registry studies have shown that testosterone therapy is associated with progressive reductions in hsCRP levels, for example the study by Yassin et al. (32). However, as these are observational studies, they should be interpreted cautiously due to the possibility of residual confounding and the absence of randomized allocation, which limits the ability to draw causal conclusions.

In contrast, Zhao et al. (2015) conducted a study of 289 younger and 4212 older Chinese participants using a separate-sample Mendelian randomization approach to mitigate reverse causation. Their results did not reveal any significant association between endogenous testosterone and systemic inflammation markers, including hsCRP. Based on these findings, the authors concluded that testosterone may not exert anti-inflammatory effects in the context of chronic diseases linked to low-grade systemic inflammation (33). These discrepancies between observational and genetic studies highlight the complexity of the relationship between testosterone and inflammation and suggest that, although testosterone’s anti-inflammatory properties may be supported by mechanistic and some clinical data, they are not yet definitively established.

In animal models, Crisostomo et al. demonstrated that testosterone administration prior to ischemic events was associated with intensified inflammatory responses in both male and female castrated rats, as evidenced by increased activation of signaling proteins p38 and SPAK/JNK, which are involved in myocardial inflammation (34).

Overall, existing studies suggest a negative correlation between testosterone and inflammatory status. Testosterone therapy has been associated with beneficial modulation of inflammatory pathways and may correspond to clinical improvement. In hypogonadal men, testosterone replacement has been linked to a 39% reduction in mortality risk, with a hazard ratio of 0.61 (95% CI: 0.42–0.88) compared to those not receiving treatment (35). Moreover, testosterone supplementation has been associated with reductions in visceral fat, fasting plasma glucose (FPG), and triglyceride levels in older men, potentially contributing to lower hsCRP levels and a reduced likelihood of cardiovascular events. Traish et al. reported a significantly lower rate of cardiovascular mortality in patients treated with testosterone undecanoate compared to those in the untreated control group (36). Finally, recent evidence indicates that long-term testosterone therapy (lasting at least six months) is consistently associated with decreased hsCRP concentrations in aging men with testosterone deficiency (37, 38).

This study has several limitations. The most significant is the potential for selection bias due to the recruitment method. Participants were volunteers referred by primary care physicians rather than selected from a random population sample. This approach may have attracted individuals who are more health-conscious or have specific pre-existing health concerns, thereby limiting the generalizability of our findings to the broader population of older men. Furthermore, the study did not collect detailed, controlled data on socio-economic status, diet, or lifestyle factors, which prevented adjustment for potential confounders. As a result, the declarative sociodemographic data that was collected may be of limited reliability. Additionally, no information was gathered regarding the specific motivation behind participants’ decisions to take part in the study. Future research should incorporate multi-marker inflammatory profiling, including cytokines such as interleukin-6 (IL-6) and tumor necrosis factor alpha (TNF-α), to provide a more comprehensive understanding of the inflammatory milieu potentially associated with testosterone deficiency syndrome in aging men.

In the conducted study, the presence of TDS was associated with elevated hsCRP levels compared to patients without testosterone deficiency. The concurrent presence of testosterone deficiency and elevated inflammatory markers may correlate with alterations in both biochemical and anthropometric parameters. It is also important to consider that hormonal changes in aging men may be influenced by lifestyle-related factors.

## 5 Conclusions

In the conducted study, the presence of TDS was associated with elevated hsCRP levels compared to patients without testosterone deficiency. The concurrent presence of testosterone deficiency and elevated inflammatory markers may correlate with changes in both biochemical and anthropometric parameters. It is important to note that age-related hormonal changes in men may also be influenced by modifiable lifestyle factors. Therefore, promoting a healthy lifestyle, regular physical activity, and a balanced diet appears essential for reducing systemic inflammation. Additionally, attention should be given to the potential compounding effects of altered testosterone levels and increased levels of pro-inflammatory cytokines associated with excessive body mass.

## Data Availability

The raw data supporting the conclusions of this article will be made available by the authors, without undue reservation.
